# Supplementary material for: Home delivery among women who had optimal ANC follow-up in Sub-Saharan Africa: A multilevel analysis
Source: PLoS One. 2023 Nov 30;18(11):e0295289. doi: 10.1371/journal.pone.0295289 (PMC10688839; doi:10.1371/journal.pone.0295289)
Supplement: Attachment — Submitted filename: Response to Reviewers.docx [file pone.0295289.s001.docx]

**Response to reviewers' comments**

PONE-D-23-19452

**Title: Prevalence and determinants of home delivery among women who had optimal ANC follow-up in Sub-Saharan Africa countries: A Multilevel Analysis of a Recent Demographic and Health Survey**

**Journal:** PLOS ONE

**Authors:** *Alebachew Ferede Zegeye, Wubshet Debebe Negash, Alemneh Tadesse Kassie, Likinaw Abebaw Wassie, Tadesse Tarik Tamir*

**Subject:** Submission of revised manuscript

Yours sincerely,

**Alebachew Ferede Zegeye (on behalf of all authors)**

University of Gondar, Gondar, Ethiopia

**Response to editor comments**

1. Please ensure that your manuscript meets PLOS ONE's style requirements, including those for file naming. The PLOS ONE style templates can be found at

https://journals.plos.org/plosone/s/file?id=wjVg/PLOSOne_formatting_sample_main_body.pdf and

<https://journals.plos.org/plosone/s/file?id=ba62/PLOSOne_formatting_sample_title_authors_affiliations.pdf>.

- **Response**: Dear editor, thank you for the recommendations. The authors ensured for the manuscript meeting PLOS ONE's style requirements.

2. We noticed you have some minor occurrence of overlapping text with the following previous publication(s), which needs to be addressed:

<https://www.frontiersin.org/articles/10.3389/fpubh.2022.862616/full>

<https://pubmed.ncbi.nlm.nih.gov/36906518/>

In your revision ensure you cite all your sources (including your own works), and quote or rephrase any duplicated text outside the methods section. Further consideration is dependent on these concerns being addressed.

- **Response**: Dear editor, thank you for the concern. Dear Editor, As you mentioned above, we have used all the literature as a source by paraphrasing and removing text that overlapped, then acknowledging accordingly. Kindly see sample references 2 and 7. Thank you.

3. Thank you for stating the following financial disclosure:

“No funding”

At this time, please address the following queries:

a) Please clarify the sources of funding (financial or material support) for your study. List the grants or organizations that supported your study, including funding received from your institution.

b)	State what role the funders took in the study. If the funders had no role in your study, please state: “The funders had no role in study design, data collection and analysis, decision to publish, or preparation of the manuscript.”

c) If any authors received a salary from any of your funders, please state which authors and which funders.

d) If you did not receive any funding for this study, please state: “The authors received no specific funding for this work.”

Please include your amended statements within your cover letter; we will change the online submission form on your behalf.

- **Response**: Dear editor, thank you for the recommendations. Comment accepted and corrected accordingly. Kindly see the amendment in our cover letter.

4. We note that you have stated that you will provide repository information for your data at acceptance. Should your manuscript be accepted for publication, we will hold it until you provide the relevant accession numbers or DOIs necessary to access your data. If you wish to make changes to your Data Availability statement, please describe these changes in your cover letter and we will update your Data Availability statement to reflect the information you provide.

- **Response**: Dear editor, thank you for the information. The authors agreed to not change the data availability statement.

5. Your ethics statement should only appear in the Methods section of your manuscript. If your ethics statement is written in any section besides the Methods, please move it to the Methods section and delete it from any other section. Please ensure that your ethics statement is included in your manuscript, as the ethics statement entered into the online submission form will not be published alongside your manuscript.

- **Response**: Dear editor, thank you for your guidance on the ethics statement of the manuscript. The authors have written the ethics statement only in the method section based on your direction. Kindly find the point on page number 7 of our revised manuscript.

6. Please include a separate caption for each figure in your manuscript

- **Response**: Dear editor, thank you for the concern. We believe that now we have addressed the issue. Kindly find the point in the caption of figures 1 and 2 in our updated manuscript.

Kind Regards,

**Response to reviewer comments**

We are very welcome for the reviews provided by the editor and reviewers of this manuscript. The comments are encouraging and the review appears to share our judgment that this study and its results are clinically important. Please see below, our detailed response to the comments. Also, kindly find the attached file of the unmarked version of the revised manuscript and the revised manuscript with track changes that are uploaded separately.

**Reviewer #1**

* The introduction in the abstract is long and needs to be shortened.

- **Response:** Dear reviewer, First and foremost, thank you for your insightful comments. We have reduced the introduction to the abstract section as per your recommendation. Kindly see the amendments in the revised manuscript on page 2, line numbers 25–30.

* The type of study should be included in the method.

- **Response:** Dear reviewer, Thank you for the concern. The type of study in the method section has been included based on your direction. Please see the point in the revised manuscript on page 4, line number 100.

***** There is no information about existing studies with a similar objective. In this case, the first preference is to conduct studies in similar geographical areas, and the second preference is to conduct studies in other parts of the world where home birth is common.

- **Response:** Dear reviewer, Thank you for your in-depth review of our manuscript. There are some studies conducted in Ethiopia with similar objectives. To avoid duplication, our interest was the second preference to conduct studies in other parts of the world (Sub-Saharan Africa), where home birth is common. So we appended the 23 sub-African countries DHS data and analysed it to investigate prevalence as well as individual and community-level factors that determine home delivery in developing countries where home delivery is expected to be high. Kindly see some of the existing studies conducted in similar geographical areas:
- Yebyo H, Alemayehu M, Kahsay A. Why do women deliver at home? Multilevel modeling of Ethiopian National Demographic and Health Survey data. PLoS One. 2015; 10(4):e0124718.
- Chernet AG, Dumga KT, Cherie KT. Home delivery practices and associated factors in Ethiopia. Journal of reproduction & infertility. 2019;20(2):102.Berhe R, Nigusie A. Magnitude of home delivery and associated factors among child bearing age mothers in Sherkole District, Benishangul Gumuz regional state-Western-Ethiopia. BMC public health. 2020; 20(1):1-7.

* What has been the shortcoming of the existing studies that the authors have investigated this relationship with the same goal?

- **Response:** Dear reviewer, we thank you for your constructive comments and concerns. Dear reviewer, the existing studies focused on individual-level variables but did not consider community-level variables during analysis, did not consider the ANC status of women, and most of the studies used single-country DHS data. Moreover, the majority of studies used primary data to investigate home delivery. However, our study included:
- Community-level determinants of home delivery
- Conduct multi-country analysis, and
- Our study participants were women who had optimal ANC follow-up (≥4 ANC follow-ups)

 * Psychological factors such as feeling less fear at home, more comfort of the mother at home and perhaps cultural factors involved in choosing to give birth at home were not seen in this study. It is necessary to consider the limitations of this study.

- **Response:** Dear reviewer, Thank you for the concern. Most of the psychological factors, including those you mentioned above, are not found in the DHS data, so we have added those variables as a limitation of our study based on your recommendation. Kindly see the amendments in the revised manuscript on page 17, line numbers 348–350.

* The results and method are very well written.

- **Response:** Dear reviewer, I appreciate the insightful remarks you made.

* It is necessary to write in the method on what basis the leveling is done and what variables each level (such as the individual level) includes? Is this stratification statistical or conceptual or both? It is necessary to address it in the introduction and in the method.

- **Response:** The comment was accepted and corrected accordingly. Dear reviewer, the data leveling of the dependent and independent variables (both individual and community-level variables) included in this study was made based on conceptual stratification using known facts and literature. Kindly see the details about “what variables are included” on page 4, line 94 and page 6, lines 125–142, and explanations about “on what basis the leveling is done” on page 6, lines 143–145.

* The results need to be reviewed by a statistician.

- **Response:** Appreciating your in-depth review of the manuscript, dear reviewer, we have consulted bio-statisticians regarding methods, statistical analysis and tools, analysis outputs (results), and writing techniques. Finally, the approaches were found to be appropriate.

* The flaw that exists in the discussion of the study is that the statistical index (odds ratio) obtained from the present study has not been compared with any other study.

- **Response:** Dear reviewer, Thank you for your recommendations. Dear reviewer if we understand you’re your concern fully, as you saw in our discussion section, there are substantial literatures compared with the present study. We compared statistical index (odds ratio) obtained from our study with the previous literatures For instance, literature 1, 22, 23, 24, 25, 26, 27, 28, 29…

***** It is necessary to review the study with conflicting results. In the following, the reason for the difference will be explained

- **Response:** We thank you for your in-depth review of the manuscript. Dear Reviewer your concerns are ultimately constructive and applicable. We didn’t find adequate studies with conflicting results, however, as per your recommendation we have been discussed some studies which have conflicting results with our findings. Kindle see amendments on page 15, lines 295 to 297.
- **Reviewer #2**

***** Title: The title is too long and seems to be carrying terms that are not relevant and can be included in the objectives e.g. if the authors remove the terms prevalence and determinants as well as the survey statement, then include these in the objectives and methodology, respectively.

- **Response:** Dear reviewer, First and foremost, thank you for your insightful constructive comments. The title of this manuscript has been reduced and the removed terms are included in the objectives and methodology sections. Please see the amendments on the revised manuscript.

* Lines 140 and 142 are a repetition of "sex of household head".

- **Response:** Dear reviewer, appreciating your in-depth review of our manuscript, we have been removed the repetition of "sex of household head" from line 142. Dear reviewer, kindly see the correction on page number 6, lines 137.

* Lines 140 and 145 are also a repetition of "wealth index".

- **Response:** Dear reviewer, thank you for the concern. The repeated “wealth index” on page 6, line 140 has been removed. Please see the correction in revised manuscript on page number 6, lines 139.

* Lines 186 and 194 have the spelling for Sub wrong and put as Sab.

- **Response:** Dear reviewer, we greatly appreciate your in-depth review of our manuscript. Lines 186 and 194 have been corrected as Sub-Saharan as per your recommendation. Kindly see the amendments in the revised manuscript on page 8, line numbers 190 and 198.

***** The font size is not uniform- especially in the following lines- 276 and 360.

- **Response**: Dear reviewer, we thank you for your constructive comments and concerns. Dear reviewer, we have corrected font size inconsistencies in our document, especially lines 276 and 360. Kindly see the corrections on page 24, line 276 and page 17, line 360

* Line 305 statement is not consistent with the information in the rest of the paragraph. Maybe the authors meant ".......associated with hospital delivery" not "....home delivery" which they put in that statement.

- **Response:** Comment accepted and amended accordingly. Dear reviewer, we would like to appreciate your in-depth review of our manuscript. As statistical output, there is consistency in this paragraph. Based on our findings, home delivery was less likely to occur among women who had previously had pregnancy complications as compared to women who did not have pregnancy complications. This means women who experienced complications can have more practical experience with life-threatening conditions than those who did not, which might encourage them to give birth at health institutions under the supervision of experts who can help them during an emergency in case pregnancy complications happen. Kindly see on the updated manuscript page 15, line 306.

* Statistical analysis: I think there is too much statistics on ORs and this tends to be very confusing and will limit readership to only those who are well conversant with detailed statistics. I propose the details on ORs and tables be trimmed and summarized to make it clearer and understandable to majority of the audience.

- **Response**: Dear reviewer, we thank you for your valuable recommendations. Dear reviewer, in multilevel modeling, there should be a total of four models. The first is the null model with no exposure variables that is used to verify the variation in the community and give evidence to evaluate random effects at the level of the community. The second model is the adjustment of the multiple variable models for individual variables, and the third model is adjusted to consider factors at the community level. In the fourth model, potential candidate variables from individual and community variables are adjusted to the outcome variable. In this sense, we have to have at least three statistical indexes (ORs) in our association table. If we trim and summarize the association tables for the purpose of making them clearer and more understandable to the audience, the meaning and details are going to be vague, so for this reason we are obliged to show all statistical indexes in our document.

Kind regards,
